# *Sox9*-Haploinsufficiency Causes Glucose Intolerance in Mice

**DOI:** 10.1371/journal.pone.0023131

**Published:** 2011-08-02

**Authors:** Claire L. Dubois, Hung Ping Shih, Philip A. Seymour, Nisha A. Patel, James M. Behrmann, Victoria Ngo, Maike Sander

**Affiliations:** Department of Pediatrics and Cellular & Molecular Medicine, University of California San Diego, La Jolla, California, United States of America; University of Bremen, Germany

## Abstract

The HMG box transcription factor Sox9 plays a critical role in progenitor cell expansion during pancreas organogenesis and is required for proper endocrine cell development in the embryo. Based on *in vitro* studies it has been suggested that Sox9 controls expression of a network of important developmental regulators, including Tcf2/MODY5, Hnf6, and Foxa2, in pancreatic progenitor cells. Here, we sought to: 1) determine whether *Sox9* regulates this transcriptional network *in vivo* and 2) investigate whether reduced *Sox9* gene dosage leads to impaired glucose homeostasis in adult mice. Employing two genetic models of temporally-controlled *Sox9* inactivation in pancreatic progenitor cells, we demonstrate that contrary to *in vitro* findings, *Sox9* is not required for Tcf2, Hnf6, or Foxa2 expression *in vivo*. Moreover, our analysis revealed a novel role for Sox9 in maintaining the expression of Pdx1/MODY4, which is an important transcriptional regulator of beta-cell development. We further show that reduced beta-cell mass in *Sox9*-haploinsufficient mice leads to glucose intolerance during adulthood. *Sox9*-haploinsufficient mice displayed 50% reduced beta-cell mass at birth, which recovered partially via a compensatory increase in beta-cell proliferation early postnatally. Endocrine islets from mice with reduced *Sox9* gene dosage exhibited normal glucose stimulated insulin secretion. Our findings show Sox9 plays an important role in endocrine development by maintaining *Ngn3* and *Pdx1* expression. Glucose intolerance in *Sox9*-haploinsufficient mice suggests that mutations in *Sox9* could play a role in diabetes in humans.

## Introduction

Both proper neogenesis of mature endocrine cells during embryonic pancreas development and the maintenance of an adequate number of functional islets during adulthood are necessary for normal glucose homeostasis. During development, endocrine and exocrine (ductal and acinar) cells of the pancreas derive from multipotent progenitor cells (MPCs) expressing the transcription factors Pdx1 [1], Ptf1a [2], Sox9 [3] and Tcf2 (Hnf1β) [4]. Furthermore, all five endocrine cell types, including the insulin^+^ beta-cells, arise from a common transient endocrine progenitor marked by expression of the transcription factor Neurogenin 3 (Ngn3) [1]. Proper allocation of MPCs to the endocrine lineage is achieved by induction of Ngn3 [5], which is tightly governed by a complex transcriptional network involving Notch signaling as well as transcription factors, such as Tcf2, Foxa2 (Hnf3β), and Hnf6. While it is well established that Pdx1 (also known as maturity onset diabetes of the young [MODY] 4 [6], [7], [8]) is required for MPC specification as well as for proper adult islet function [9], [10], [11], Pdx1 has also recently been shown to regulate *Ngn3* directly in cooperation with Hnf6 [12].

We have shown previously that the MPC marker and maintenance factor *Sox9* [3], [13], [14] governs endocrine development in a dosage-dependent manner [14]. Pancreas-specific *Sox9*-haploinsufficiency in *Pdx1-Cre*; *Sox9^+/flox^* (*Sox9^+/Δpan^*) mice results in a 50% decrease in both Ngn3^+^ endocrine progenitors and consequently, endocrine cells at birth [14]. While reduced in numbers, immunohistochemical analysis of beta-cell markers suggested that beta-cells are properly differentiated in *Sox9*-haploinsufficient mice. Though Sox9 has been shown to bind the *Ngn3* promoter in embryonic pancreas *in vivo* [14], *in vitro* studies have implied that it also regulates *Hnf6*, *Tcf2*, and *Foxa2* and is therefore critical for activating and/or maintaining an entire network of endocrine differentiation genes [15]. However, because early deletion of *Sox9* in pancreatic progenitors results in developmental arrest prior to the onset of endocrine cell differentiation [13], the role of Sox9 in regulating key endocrine differentiation genes *in vivo* has not been studied. By employing temporally controlled *Sox9* inactivation strategies in mice, we thus sought to determine which components of the pancreatic transcriptional network are regulated by Sox9 *in vivo*.

MODY is a disease caused by mutations in autosomal dominant genes: MODY1–5 result from mutations in *Hnf4α*, *glucokinase*, *Hnf1α*, *Pdx1*, and *Tcf2*, respectively. Most MODY genes are expressed in the mature islet; their downregulation is associated with loss of islet function, manifesting in diabetes [9], [16], [17], [18]. Tcf2 however, is not expressed in beta-cells. Mirroring the expression pattern of Sox9, it is expressed in the MPC population, then confined to ductal and centroacinar cells in adult pancreas [3], [19]. Thus, MODY can occur not only as a result of mutations in genes expressed in adult beta-cells, but also, due to mutations in genes expressed in the progenitors from which they arise. Tcf2 exemplifies that defects in the embryonic development of beta-cells can manifest in diabetes later in life. On the basis of the findings that: 1) *Sox9*-haploinsufficient mice are born with half the normal complement of pancreatic endocrine cells [14]; 2) Sox9 colocalizes with Tcf2 in embryonic and adult pancreas [3], and 3) Sox9 regulates *Tcf2 in vitro* [15], we sought to explore whether reduced Sox9 activity could result in a diabetic phenotype during adulthood.

Here, we identify a novel role for Sox9 in the regulation of the transcriptional network upstream of Ngn3, specifically in the maintenance of Pdx1 (MODY4) expression. Contrary to previous *in vitro* studies we find that Sox9 is dispensable for expression of Tcf2, Hnf6, and Foxa2 in the developing pancreas. Additionally, by characterizing the adult phenotype of *Sox9*-haploinsufficient mice, we show that reduction of *Sox9* gene dosage causes glucose intolerance. *Sox9*-haploinsufficient mice did not progress to overt diabetes, likely owing to a compensatory postnatal increase in beta-cell proliferation and mass observed in *Sox9^+/Δpan^* mice.

## Methods

### Ethics Statement

All animal experiments described herein were approved by the University of California, Irvine and San Diego Institutional Animal Care and Use Committees (protocol numbers 2001–2420 and S08215, respectively).

### Mouse Strains and Husbandry

*Pdx1-Cre; Sox9^flox/flox^* (*Sox9^Δpan/Δpan^*) and *Sox9^+/Δpan^* mice were generated and maintained as previously described [14]. *Gt(ROSA)26Sor^tm1(cre/Esr1)Nat^/J* (hereon abbreviated to *R26CreTM*) mice [20] were obtained from Jackson Laboratory (JAX) and maintained on a C57BL/6J background before breeding to *Sox9^flox/flox^* mice. In all experiments, Cre^−^ littermates served as controls. Embryos were harvested from timed matings in which noon on the day of vaginal plug appearance was considered as e0.5. *Sox9*-ablation in *R26CreTM*; *Sox9^flox/flox^* (*Sox9^Δe13/Δe13^*) and *Sox9^+/Δe13^* mice was induced by intraperitoneal (i.p.) injection of 3 mg/40 g body weight tamoxifen (Sigma) dissolved in corn oil (Sigma) into e12.5 or e14.5 pregnant dams. Since recombination in this system occurs within 6 hours of tamoxifen administration [21], [22], *R26CreTM; Sox9^flox/flox^* mice will be denoted as *Sox9* ^Δe13/Δe13^ or *Sox9* ^Δe15/Δe15^, respectively. For BrdU labeling of 2-week-old mice, 50 mM BrdU (Sigma) was injected i.p. 6 hours before sacrifice. In 6-week-old mice, BrdU labeling was achieved by administration of 1 mg/mL BrdU in the drinking water for one week before sacrifice. High-fat diet-fed mice were fed a 60% high-fat diet from Bio-Serv (F3282).

### Histological Analysis and Beta-Cell Mass Measurements

Tissue preparation, immunochemistry, imaging, and morphometric analysis were performed as previously described [14]. Beta-cell mass was calculated as follows: (insulin^+^ area/total pancreatic area) multiplied by pancreatic weight. For beta-cell proliferation measurements, BrdU and insulin co-positive cells were counted and expressed relative to total insulin^+^ cells. A minimum of 50 islets were analyzed per animal. At least 3 mice were analyzed for each experimental group.

The following primary antibodies were used at the given dilutions: rabbit anti-Sox9 (Chemicon), 1∶1000; goat anti-Sox9 (Santa Cruz), 1∶100; guinea pig anti-Pdx1 (kindly provided by C. V. E. Wright, Vanderbilt University, Nashville TN), 1∶10,000; rabbit anti-Hnf6 (Santa Cruz), 1∶200; goat anti-Spp1 (R&D Systems), 1∶1000; goat anti-Foxa2 (Santa Cruz), 1∶200; goat anti-Tcf2 (Santa Cruz), 1∶100; rat anti-E-cadherin (Sigma), 1∶1000; guinea pig anti-insulin (DAKO), 1∶1000; mouse anti-glucagon (DAKO), 1∶10,000 and mouse anti-BrdU (Chemicon), 1∶200. Secondary antibodies were diluted 1∶2000 (Jackson ImmunoResearch or Invitrogen).

### Quantitative RT-PCR and Western Blot

At e15.5, RNA was extracted from pancreata of nine embryos per experimental group and three pancreata per genotype were pooled. Each PCR was run in triplicate. RNA isolation, cDNA synthesis and quantitative (q)RT-PCR were performed and analyzed as previously described [14].

Primers (5′-3′) used were: *Sox9* forward: AGACTCACATCTCTCCTAATGCT and reverse: ACGTCGGTTTTGGGAGTGG; *Foxa2* forward: AGGCACTGCGCTTCACTCC and reverse: CTCATTCCAGCGCCCACATAG; *Ngn3* forward: AATGATCGGGAGCGCAATCG and reverse: CGCAGGGTCTCGACCTTTG; *Pdx1* forward: GATGAAATCCACCAAAGCTCA and reverse: AGAATTCCTTCTCCAGCTCCA; *Hnf6* forward: GGCAACGTGAGCGGTAGTTT and reverse: TTGCTGGGAGTTGTGAATGC; *Tcf2* forward: GCCTGAACCAATCCCACCTC and reverse: TGACTGCTTTTGTCTGTCATGT.

Protein for Western blots was obtained, processed, transferred to nitrocellulose, and incubated with primary antibody as previously described [14]. Pancreata from 3 mice were pooled per sample. Signal was detected and quantified using the Odyssey infrared imaging system and reagents (LI-COR Technologies). Values were calculated relative to GAPDH expression.

### Metabolic Assays

Intraperitoneal glucose tolerance tests (IPGTTs) were performed on mice after 16 hours of fasting. Mice were injected i.p. with 1.5 mg/g body weight dextrose solution and their blood glucose was measured at the outset as well as 20, 40, 60, 90, and 120 minutes post-challenge. For *in vitro* glucose stimulated insulin secretion (GSIS) assays, islets were isolated as previously described [14] and cultured overnight at 37°C in RPMI 1640 (Mediatech) supplemented with 4.8 mM D-glucose (Fisher), 10 mM HEPES (Sigma), 2 mM glutamine (Gibco), 1 mM sodium pyruvate (Sigma), 10% FBS (Sigma) and 1% penicillin/streptomycin (Mediatech). Sets of 30 islets were incubated in 500 µl KRBH buffer [23] containing 2.8 mM D-glucose for 1 hour at 37°C. Buffer was replaced with KRBH containing 16.7 mM D-glucose for an additional 2-hour incubation. Buffer was collected and islets were sonicated in acid ethanol. Insulin content was separately determined in the supernatant and the islet cell fraction and the percentage of secreted insulin calculated as: 100*(secreted insulin/secreted insulin+islet cell insulin), as previously described [24]. Whole pancreas extracts were obtained by homogenizing whole pancreata in acid ethanol, incubating overnight at 4°C, and harvesting the supernatant. Serum was collected from the blood of 16-hour fasted mice. In all cases, insulin content was determined by mouse insulin ELISA (ALPCO Diagnostics).

### Statistical Analysis

All values are shown as mean ± standard error of the mean (S.E.M.); *p* values were calculated using unpaired two-tailed Student's t test; *p*<0.05 was considered significant.

## Results

### Sox9 expression parallels that of a network of factors upstream of *Ngn3*

Previously, we showed that *Sox9^+/Δpan^* embryos exhibit a 50% reduction in the number of Ngn3^+^ endocrine progenitors and consequently, all mature endocrine cell types [14]. However, it remains unclear why endocrine cells are reduced in *Sox9^+/Δpan^* embryos and how Sox9 orchestrates endocrine cell development in conjunction with other transcriptional regulators that control endocrine cell differentiation. It has been reported that Sox9 regulates the transcription factors *Hnf6*, *Tcf2*, and *Foxa2*, in the mPAC pancreatic tumor cell line [15]. However, it has not been studied whether Sox9 controls the expression of these endocrine differentiation genes during development. Therefore, to test whether this regulation occurs *in vivo*, we first examined whether the expression domain of Sox9 coincides with those of these transcription factors in MPCs.

**Figure 1:**
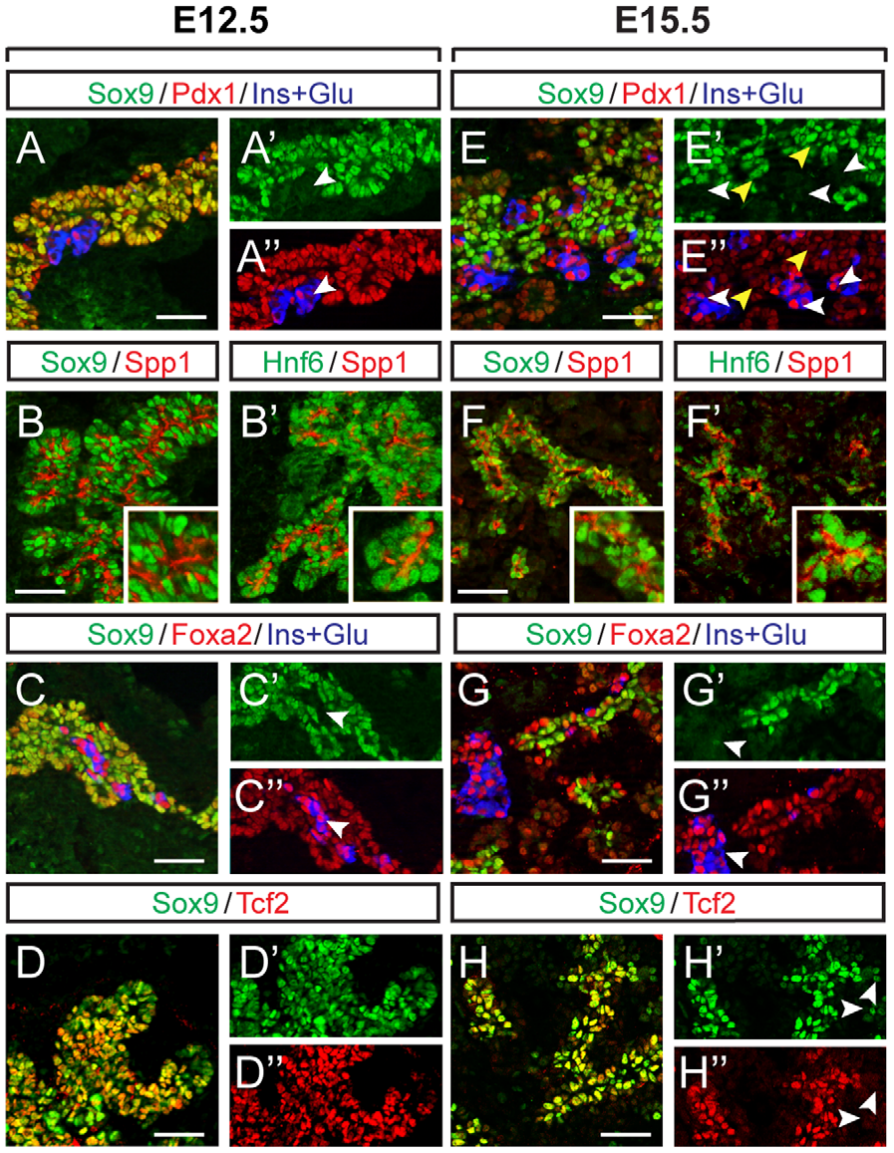
Sox9 is co-expressed with Hnf6, Foxa2, Tcf2, and Pdx1 in progenitor cords of the developing pancreas. Immunofluorescence staining of e12.5 (**A–D**) and e15.5 (**E–H**) *wild-type* pancreata reveals extensive overlap of the Sox9^+^ domain with Hnf6 (**B, F**), Foxa2 (**C, G**), and Tcf2 (**D, H**). (**B, F**) Osteopontin (Spp1), which marks the apical surface of cells in the lumen of the epithelial cords, is used as a reference for the Sox9 and Hnf6 expression domains. (**A**) Sox9 is widely coexpressed with Pdx1 throughout the pancreatic epithelium at e12.5. Pdx1^+^/Sox9^−^ cells at this stage are insulin/glucagon^+^ (ins+glu) (**A′-A″**, white arrowheads). (**E**) At e15.5, Sox9^+^ cells are found restricted to the inner epithelial layer and are weakly Pdx1^+^ (**E′-E″**, yellow arrowheads). At this time, differentiated cells expressing high levels of Pdx1 are insulin/glucagon^+^, but Sox9^−^ (**E′-E″**, white arrowheads). Similarly, Foxa2^+^/Sox9^−^ cells producing insulin/glucagon are present at e12.5 (**C′-C″**, white arrowheads), as well as at e15.5 (**G′-G″**, white arrowheads). (**D**) At e12.5, Sox9 and Tcf2 widely colocalize throughout the pancreatic epithelium. (**H**) By e15.5, some cells that are Sox9^+^/Tcf2^−^ are visible at the distal tips of the epithelial cords (**H′-H″**, white arrowheads). Scale bars: 50 µm.

At e12.5, when the pancreatic epithelium predominantly comprises undifferentiated progenitors, Sox9 is widely co-expressed with Pdx1 (**Fig. 1A-A″**). At this stage, Sox9^+^ cells are also intimately associated with cells expressing osteopontin (Spp-1), which, like mucin-1, marks the apical aspect of cells lining the forming luminal network within a still-stratified epithelium [25] (**Fig. 1B**). Paralleling the expression domain of Sox9, Hnf6 similarly marks cells lining the Spp-1-delineated epithelial lumen (**Fig. 1B-B′**). At e12.5, Sox9 also widely co-localizes with Foxa2 (**Fig. 1C-C″**) and Tcf2 (**Fig. 1D-D″**). Some cells already expressing glucagon and/or insulin with elevated Foxa2 levels but little or no Sox9 were also present (**Fig. 1C-C″**).

By e15.5, during the major window of pancreatic differentiation (termed the secondary transition), Sox9 becomes exclusively restricted to the monolayered, polarized epithelial “cords” (**Fig. 1E–H″**), from which Ngn3^+^ endocrine progenitors and their descendents as well as mature ductal cells arise [3], [4]. In the progenitor epithelium, Sox9 co-localized with Pdx1 (**Fig. 1E′,E″**, yellow arrowheads). Notably, Sox9^+^ progenitors displayed lower levels of Pdx1 expression than the Sox9^−^ hormone^+^-cells that have delaminated from the progenitor cell epithelium (**Fig. 1E′,E**″, yellow *versus* white arrowheads) [26]. Sox9^+^ progenitors also expressed Foxa2 at e15.5 (**Fig. 1G-G″**). As previously reported [4], progenitor cells of the e15.5 epithelial cords were additionally marked by Tcf2 (**Fig. 1H**). However, at the distal tips of the lumens, we consistently observed cells that expressed high levels of Sox9, but little or no Tcf2 (**Fig. 1H′,H″**, arrowheads). These distal Sox9^+^ cells presumably constitute acinar progenitors [3].

Together, this expression analysis shows that Sox9^+^ progenitors express Pdx1, Hnf6, Foxa2, and Tcf2 during the major period of endocrine cell differentiation.

### *Sox9* is required for the maintenance of Pdx1

**Figure 2:**
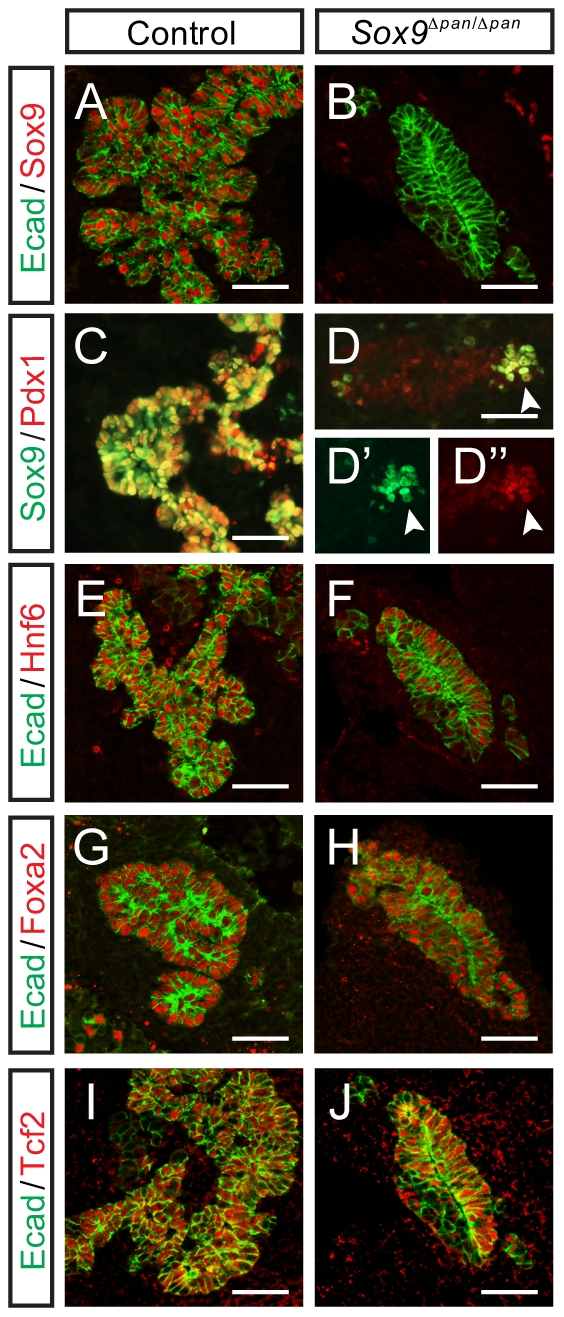
Pancreas-specific *Sox9*-deletion results in loss of Pdx1 by e12.5. Immunofluorescence staining of control (**A**, **C**, **E**, **G**, **I**) and *Sox9^Δpan/Δpan^* pancreata (**B**, **D**, **F**, **H**, **J**) at e12.5. *Pdx1-Cre*-mediated *Sox9-flox* recombination efficiently ablates Sox9 (**B**), resulting in pancreatic hypoplasia (**B**, **D**, **F**, **H**, **J**). (**A**, **B**, **E–J**) E-cadherin (Ecad) was used to visualize the Sox9^−^ pancreatic epithelium. While loss of Sox9 does not affect expression of Hnf6 (**F**), Foxa2 (**H**), or Tcf2 (**J**), Pdx1 expression is reduced (**D**). (**D**) Mosaic recombination results in an unrecombined population of Sox9^+^ cells, which retains high levels of Pdx1 (white arrowheads). Scale bars: 50 µm.

Next, we examined whether *Sox9* regulates the expression of the transcription factors Pdx1, Hnf6, Foxa2, and Tcf2 in the pancreatic progenitor epithelium. We have shown that *Pdx1-*Cre-mediated inactivation of both *Sox9^flox^* alleles leads to pancreatic hypoplasia (**Fig. 2B,D,F,H,J**; [13]). Although we saw no obvious decrease in Pdx1 expression at e10.5 in *Sox9^Δpan/Δpan^* pancreata [13], by e12.5, there was a striking decrease in Pdx1 immunofluorescence intensity in *Sox9-*deleted cells (**Fig. 2C,D**). In contrast to our earlier analysis [13], some mosaicism was apparent in the deletion of *Sox9* by *Pdx1*-*Cre*. As such, robust Pdx1 expression was maintained only in those progenitor cells that retained Sox9 (**Fig. 2D**, arrowheads). Together, the retention of Pdx1 in Sox9^+^ cells and the gradual loss of Pdx1 in *Sox9*-deleted cells suggest that Sox9 is required for the maintenance of Pdx1 expression in pancreatic progenitors. Contrastingly, at e12.5, the expression of Hnf6, Foxa2, and Tcf2 appeared unaffected by *Sox9* deletion (**Fig. 2E–J**).

It has been proposed that Sox9 interacts with Hnf6, Foxa2, and Tcf2 in a transcriptional network that stabilizes pancreatic progenitor gene expression, but also cooperates in activating Ngn3, which disrupts the network in order to drive endocrine differentiation [15]. To investigate whether Sox9 differentially regulates these transcription factors during the major period of endocrine cell neogenesis *in vivo*, we examined the expression of Hnf6, Foxa2, and Tcf2, as well as Pdx1 in *Sox9*-deficient pancreas at e15.5. However, the acute pancreatic hypoplasia resulting from *Pdx1-Cre*-mediated *Sox9* ablation precluded us from dissecting later roles of *Sox9* in pancreatic differentiation. To overcome this obstacle, we utilized a ubiquitously expressed tamoxifen-inducible Cre line, *R26CreTM*, to ablate *Sox9* after e12.5 and then examine the effects at e15.5 in *Sox9* ^Δe13/Δe13^ mice.

**Figure 3:**
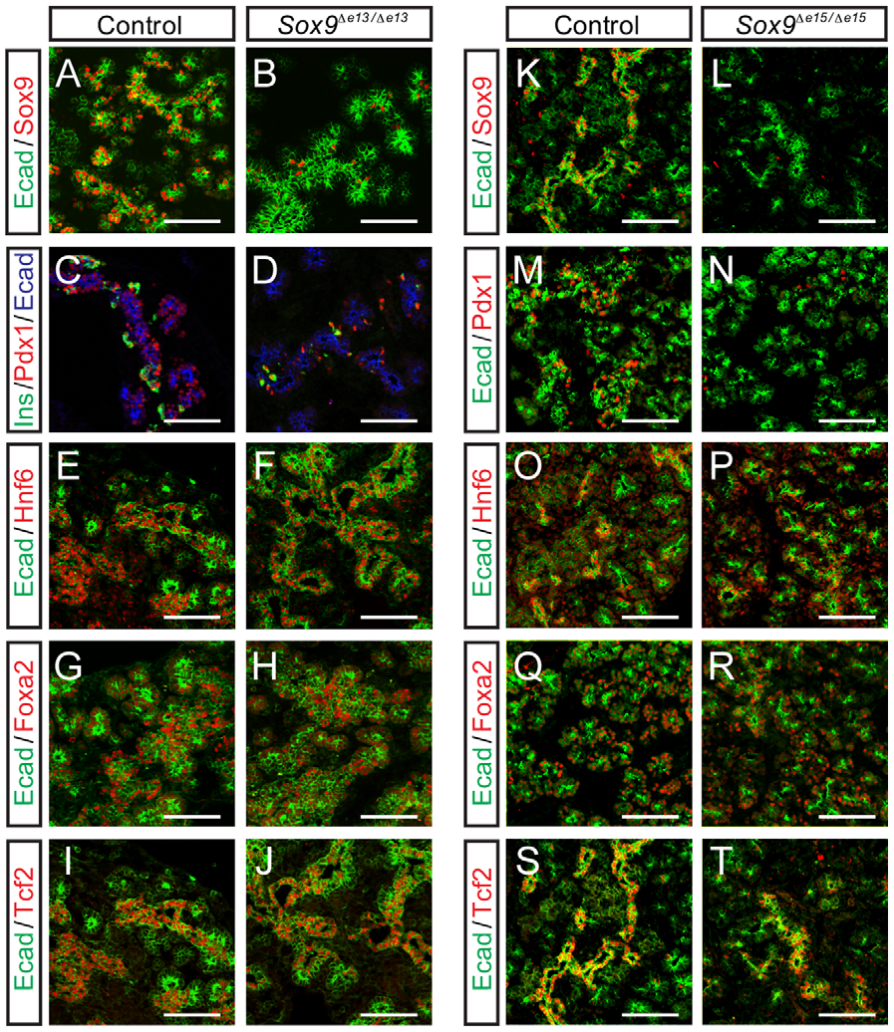
*Sox9* deletion during endocrine cell neogenesis results in reduced numbers of Pdx1^+^ cells. Immunofluorescence staining of control (**A**, **C**, **E**, **G**, **I**, **K**, **M**, **O**, **Q**, **S**) and *R26-CreER; Sox9^flox/fox^* embryos (**B**, **D**, **F**, **H**, **J**, **L**, **N**, **P**, **E**, **T**) injected with tamoxifen at e12.5 and analyzed at e15.5 (*Sox9^Δe13/Δe13^*) or injected with tamoxifen at e14.5 and analyzed at e18.5 (*Sox9^Δe15/Δe15^*). Sox9 expression is lost in the majority of E-cadherin^+^ (Ecad^+^) epithelial cells (**B, L**). Deletion of *Sox9* results in decreased expression of Pdx1 (**D, N**) as well as a reduction in the number of insulin^+^ (Ins^+^) cells, while Hnf6 (**F, P**), FoxA2 (**H, R**), and Tcf2 (**J, T**) expression is unaffected. Scale bar: 50 µm.

*R26CreTM*-mediated deletion of *Sox9* by intraperitoneal administration of tamoxifen at e12.5 in pregnant dams resulted in efficient ablation of Sox9 protein in ∼90% of the luminal epithelial cells comprising the Sox9^+^ domain at e15.5 (**Fig. 3A,B**). Since *Sox9* was inactivated after the period of rapid progenitor cell expansion, overall pancreatic organ size and morphogenesis were not significantly affected in *Sox9* ^Δe13/Δe13^ mice at e15.5 (**Fig. 3A–J** **; data not shown**). Mirroring our earlier observation of Pdx1 loss following *Pdx1-Cre*-mediated *Sox9* deletion (**Fig. 2C–D**), immunofluorescence analysis revealed a dramatic decrease in the number of Pdx1^+^ cells at e15.5 in *Sox9^Δe13/Δe13^* pancreata (**Fig. 3C,D**). This decrease was predominantly observed in the luminal epithelial cells and was accompanied by a marked reduction in the number of newly-formed Pdx1^+^/insulin^+^ cells (**Fig. 3D**). Since Pdx1 is critical for endocrine cell differentiation and regulates expression of the endocrine differentiation factor Ngn3 [12], this finding suggests that Sox9-dependent regulation of Pdx1 expression in the progenitor cell epithelium after e13.5 is critical for the major wave of beta-cell differentiation that occurs between e14 and birth. While *Sox9* deletion had a profound effect on Pdx1 expression, the expression of Hnf6, Foxa2, and Tcf2 appeared unaffected by the loss of *Sox9* (**Fig. 3E–J**). To determine whether Sox9 might control the expression of Hnf6, Foxa2, Tcf2, and Pdx1 at later time points, when Sox9 becomes restricted to the pancreatic ducts (**Fig. 3K**), we induced *Sox9* deletion by injecting pregnant dams with tamoxifen at e14.5 and analyzed the embryos at e18.5 in *Sox9* ^Δe15/Δe15^ mice. Similar to the inactivation performed at e12.5, *R26CreTM*-mediated deletion of *Sox9* by tamoxifen injection at e14.5 resulted in efficient ablation of Sox9 protein (**Fig. 3K,L**). At e18.5, Pdx1 expression is largely restricted to the endocrine cell compartment (**Fig. 3M**; [26]). *Sox9* deletion resulted in a noticeable reduction of Pdx1^+^ cells, suggesting that Sox9 continues to be required for endocrine cell formation at later stages of development (**Fig. 3N**). This finding is consistent with the observation that Sox9^+^ cells give rise to new endocrine cells until birth [3]. Mirroring our results obtained in embryos after *Sox9* deletion at earlier time points, ablation of *Sox9* during late embryogenesis did not affect the expression of Hnf6, Foxa2, and Tcf2 (**Fig. 3O–T**).

**Figure 4:**
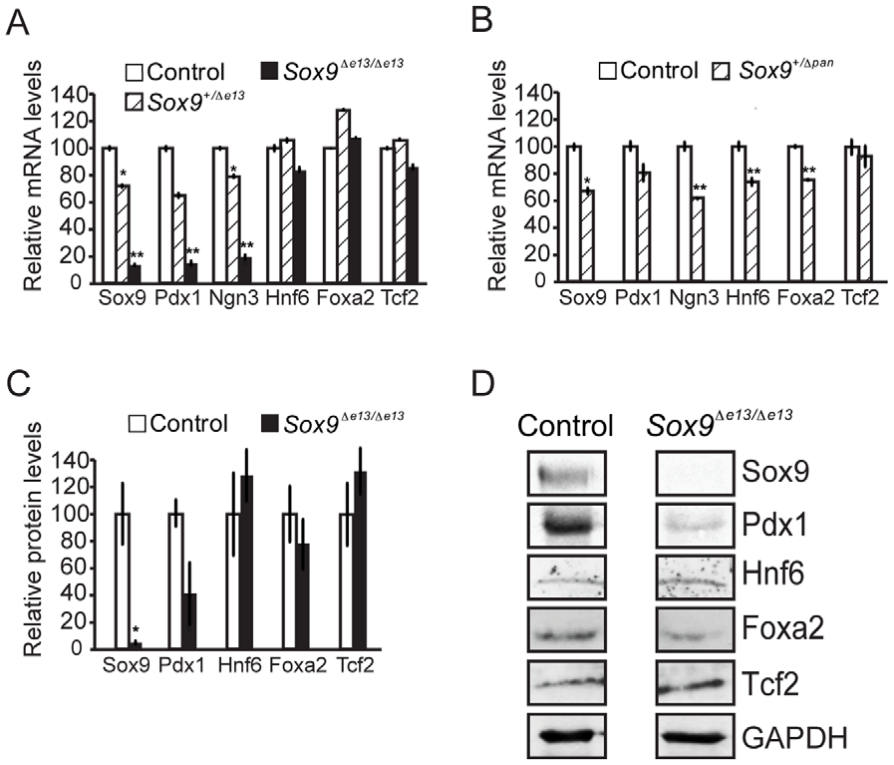
Sox9 regulates *Pdx1* mRNA and protein expression. Quantitative RT-PCR of pancreata from *Sox9^flox/fox^* or *Sox9^+/fox^* embryos injected with tamoxifen at e12.5 and analyzed at e15.5 (*Sox9^Δe13/Δe13^* or *Sox9^+/Δe13^*) (**A**; *n* = 9) shows reduced levels of *Pdx1* and *Ngn3*, but not of *Hnf6*, *FoxA2*, or *Tcf2*. In *Sox9^+/Δpan^* pancreata after *Pdx1-Cre*-mediated recombination of one *Sox9-flox* allele (**B**; *n* = 9), *Pdx1*, *Ngn3*, *Hnf6*, and *FoxA2* mRNA levels are reduced at e15.5. Western Blot analysis (**C**, **D**; *n* = 4) shows diminished Pdx1 expression, but not Hnf6, Foxa2, or Tcf2 expression in pancreata from *Sox9^Δe13/Δe13^* embryos at e15.5. Values are shown as mean ± S.E.M.; * = *P*<0.05; ** = *P*<0.01.

Quantitative RT-PCR analysis confirmed the requirement of Sox9 for Pdx1 expression, revealing a significant, dose-dependent decrease in *Pdx1* transcript levels with progressive loss of *Sox9* in pancreata of *Sox9^+/+^*→*Sox9^+/Δe13^*→*Sox9^Δe13/Δe13^* mice at e15.5 (**Fig. 4A**). Likewise, and consistent with our findings in *Sox9^+/Δpan^* mice [14] (**Fig. 4B**), *Ngn3* mRNA levels were dependent upon *Sox9* gene dosage (**Fig. 4A,B**). Reflecting the reduction in mRNA levels, whole pancreata of e15.5 *Sox9^Δe13/Δe13^* mice also exhibited a 60% reduction in Pdx1 protein levels compared with those of control littermates (**Fig. 4C,D**). Concordant with the results of our immunofluorescence analysis (**Fig. 3E–J**), Hnf6, Foxa2, and Tcf2 transcript and protein levels at e15.5 were unaffected by deletion of *Sox9* at ∼e13 (**Fig. 4A,C,D**). Unlike *Sox9^+/Δ^* ^e13^ mice (**Fig. 4A**), *Sox9^+/Δpan^* mice displayed a slight, but significant reduction in *Hnf6* and *FoxA2* mRNA levels at e15.5 (**Fig. 4B**).

Our findings reveal that Sox9 is crucially required for maintaining Pdx1 and Ngn3 expression in the pancreatic progenitor cell epithelium during the major wave of endocrine cell differentiation. Since Pdx1 has been shown to regulate the expression of *Ngn3* and, in turn, beta-cell formation [12], Sox9-dependent regulation of *Pdx1* is a critical component of the transcriptional network governing endocrine and beta-cell development. However, as *Pdx1* levels are not affected by *Sox9*-halpoinsufficiency, direct regulation of *Ngn3* by Sox9 appears to be the critical factor that accounts for reduced endocrine cell mass in pancreata of *Sox9*-haploinsufficient mice at birth. Importantly, the fact that Hnf6, Foxa2, and Tcf2 expression were not affected by deletion of *Sox9* argues strongly that, contrary to the *in vitro* findings of Lynn et al. [15], Sox9 does not regulate these upstream regulators of Ngn3 expression during pancreas organogenesis.

### Pancreatic *Sox9* haploinsufficiency results in glucose intolerance

**Figure 5:**
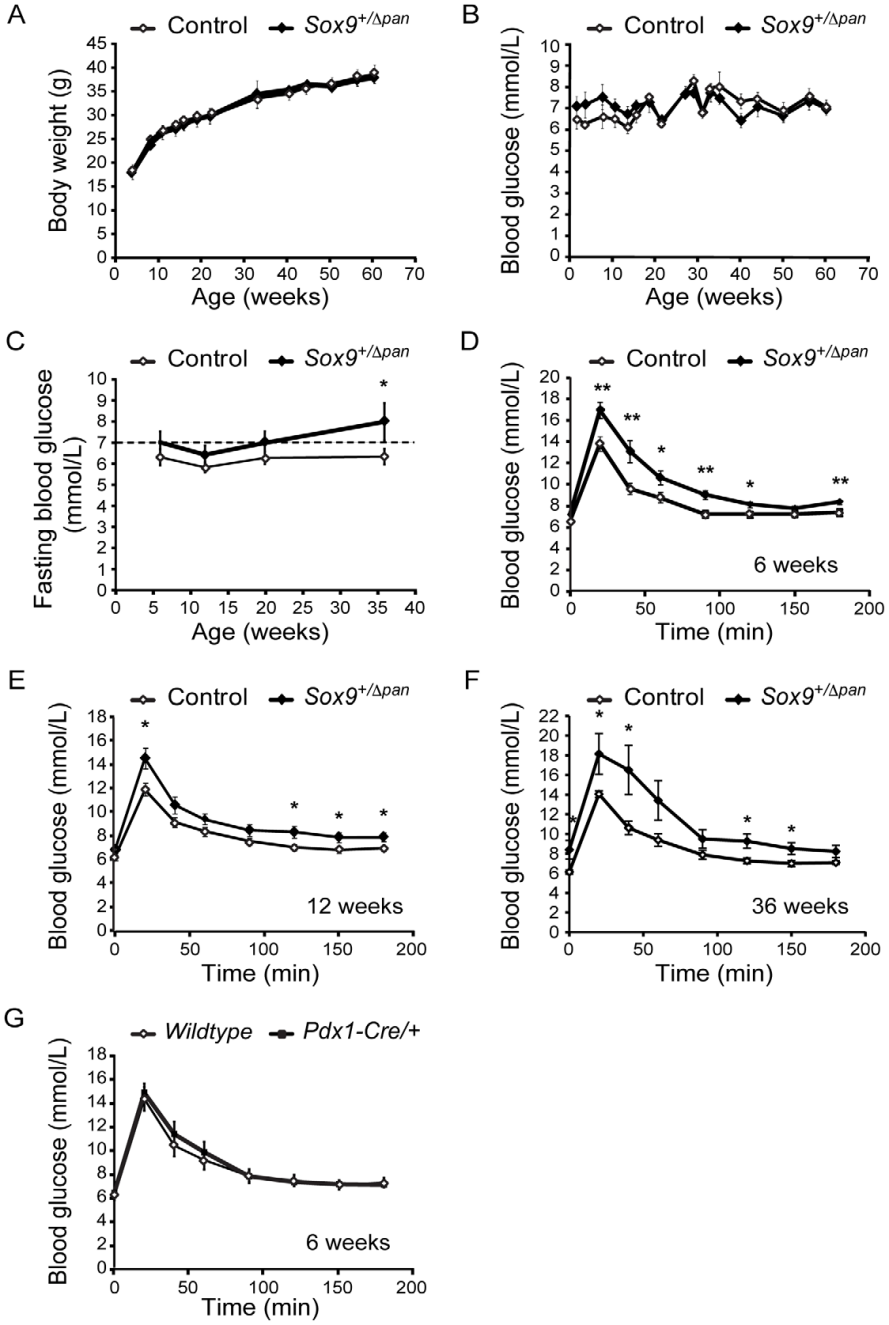
*Sox9*-haploinsufficient mice are glucose-intolerant. Body weight (**A**), random fed blood glucose levels (**B**), and fasting blood glucose levels (**C**) do not significantly differ in *Sox9^+/Δpan^* and control mice (*n* = 10), except for fasting blood glucose levels in *Sox9^+/Δpan^* mice at 36 weeks of age. (**C**) The dashed line indicates the threshold at which blood glucose levels are considered diabetic. Following an intraperitoneal glucose challenge, *Sox9^+/Δpan^* mice exhibit elevated blood glucose levels compared to control mice at 6 weeks (**D**; *n* = 20), 12 weeks (**E**; *n* = 18), and 36 weeks (**F**; *n* = 8) of age. (**G**) Blood glucose levels in response to an intraperitoneal glucose challenge are similar in *Pdx1-Cre* mice and *wild-type* littermates (*n* = 11). Values are shown as mean ± S.E.M.; * = *P*<0.05; ** = *P*<0.01.

Previous studies demonstrating that haploinsufficiency for regulators of beta-cell development can manifest in MODY-type diabetes [19], [27] led us to next examine whether *Sox9^+/Δpan^* mice display a diabetic phenotype in adulthood. To ascertain the physiological effects of pancreatic *Sox9*-haploinsufficiency in adulthood, we monitored the body weight and blood glucose levels of *ad libitum*-fed *Sox9^+/Δpan^* and Cre^−^ control mice over a 60-week period. We found that, over the period of study, *Sox9^+/Δpan^* mice showed no difference in body weight (**Fig. 5A**), nor random-fed blood glucose levels (**Fig. 5B**) compared to littermate controls. However, while there was also no significant difference between fasted blood glucose levels of *Sox9^+/Δpan^* mice and control siblings, *Sox9^+/Δpan^* mice tended to exhibit higher blood glucose levels which in time progressed to diabetic levels (>7 mmol/l in the fasted state, as indicated by the broken line; **Fig. 5C**). Consistent with developing glucose intolerance, by six weeks of age, blood glucose levels were significantly elevated in *Sox9^+/Δpan^* mice compared with control littermates following an acute glucose challenge (**Fig. 5D**). Glucose intolerance persisted in *Sox9^+/Δpan^* mice at 12 weeks of age and later time points (**Fig. 5E,F**).

In order to confirm that the glucose intolerance observed in *Pdx1-Cre; Sox9^+/flox^ versus Sox9^+/flox^* control mice was not attributable to extraneous effects of the *Pdx1-Cre* transgene as reported in the *RIP-Cre* mouse line [28], we assayed glucose tolerance in *Pdx1-Cre versus wild-type* siblings on a *Sox9^+/+^* background. The absence of any difference in blood glucose levels between the two genotypes in response to a glucose challenge (**Fig. 5G**) shows that the *Pdx1-Cre* transgene itself exerts no influence on glucose tolerance in the current studies. Thus, pancreatic *Sox9*-haploinsufficiency results in glucose intolerance.

### High-fat diet causes fasting hyperglycemia in *Sox9*-haploinsufficient mice

**Figure 6:**
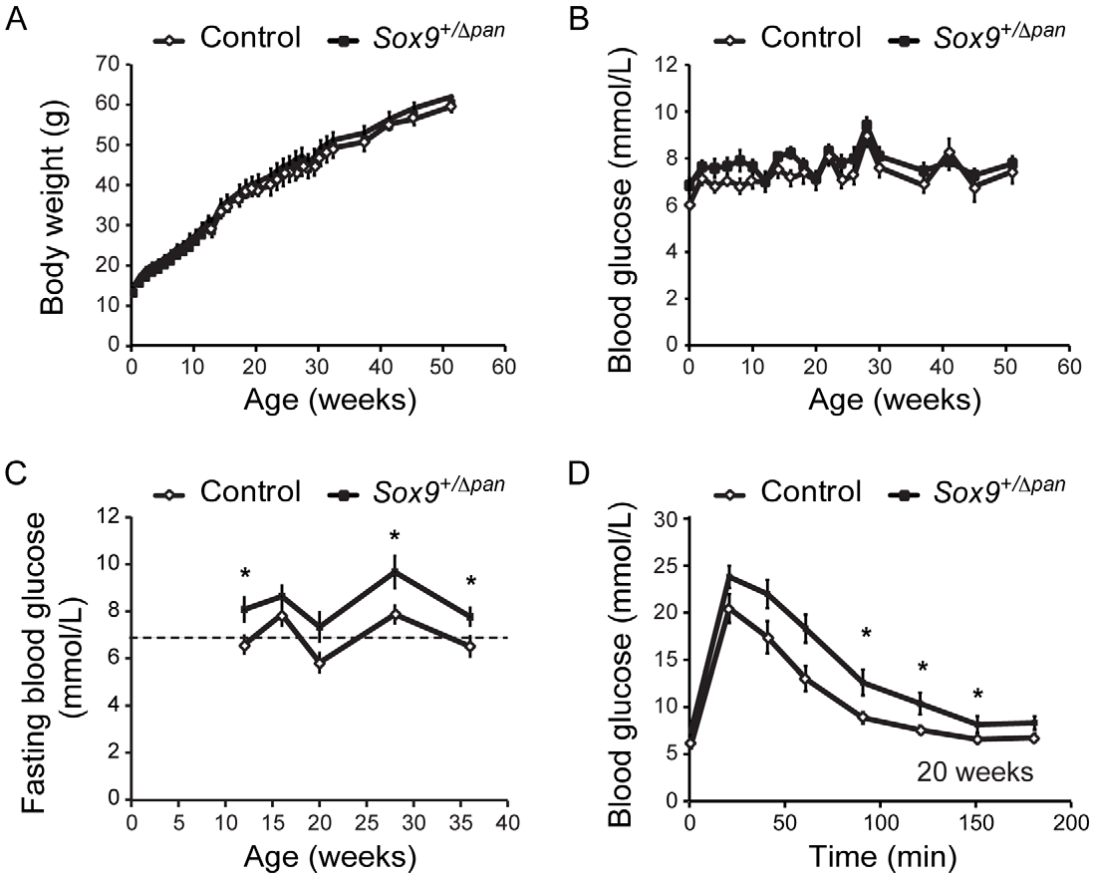
High-fat diet induces fasting hyperglycemia in *Sox9*-haploinsufficient mice. *Sox9^+/Δpan^* mice and control littermates were fed a 60% high-fat diet over 50 weeks. Body weight (**A**) and random fed blood glucose levels (**B**) do not significantly differ in *Sox9^+/Δpan^* and control mice (*n* = 14). (**C**) Fasting blood glucose levels are significantly higher in *Sox9^+/Δpan^* than control mice (*n* = 14). The dashed line indicates the threshold at which blood glucose levels are considered diabetic. (**D**) Following an intraperitoneal glucose challenge, *Sox9^+/Δpan^* mice exhibit elevated blood glucose levels compared to control mice (*n* = 14). Values are shown as mean ± S.E.M.; * = *P*<0.05.

Though the endocrine function of the reduced beta-cell complement in *Sox9^+/Δpan^* pancreata was sufficiently high to maintain normogycemia under basal conditions, we postulated that *Sox9*-haploinsufficiency might impair the ability to withstand additional metabolic stress. To test this, we maintained *Sox9^+/Δpan^* and littermate control mice on a 60% high-fat diet and monitored body weight and *ad libitum* fed blood glucose levels. Throughout the course of one year on this diet, no significant difference was detected in either body weight or blood glucose levels between *Sox9*-haploinsufficient and control siblings (**Fig. 6A,B**). However, fasting blood glucose levels in *Sox9^+/Δpan^* mice were significantly elevated over those of control animals, attaining levels considered diabetic (**Fig. 6C**). Concordantly, when high-fat diet-fed mice were subjected to intraperitoneal glucose challenge, *Sox9^+/Δpan^* mice displayed significantly elevated blood glucose levels over those of control littermates (**Fig. 6D**), mirroring the findings in normal diet-fed *Sox9^+/Δpan^* mice. Thus, the additional metabolic demand/stress of a high-fat diet mildly exacerbated the metabolic defect resulting from pancreatic *Sox9*-haploinsufficiency.

### Compensatory postnatal beta-cell proliferation in *Sox9*-haploinsufficient mice

Given that heterozygous pancreatic deletion of *Sox9* results in a two-fold decrease in beta-cell mass at birth [14], we were surprised to find that glucose homeostasis in these mice was only mildly impaired. Therefore, we sought to characterize how *Sox9*-haploinsufficient mice lacking half the usual complement of beta-cells are able to remain relatively normoglycemic, even with age and under increased metabolic stress.

**Figure 7:**
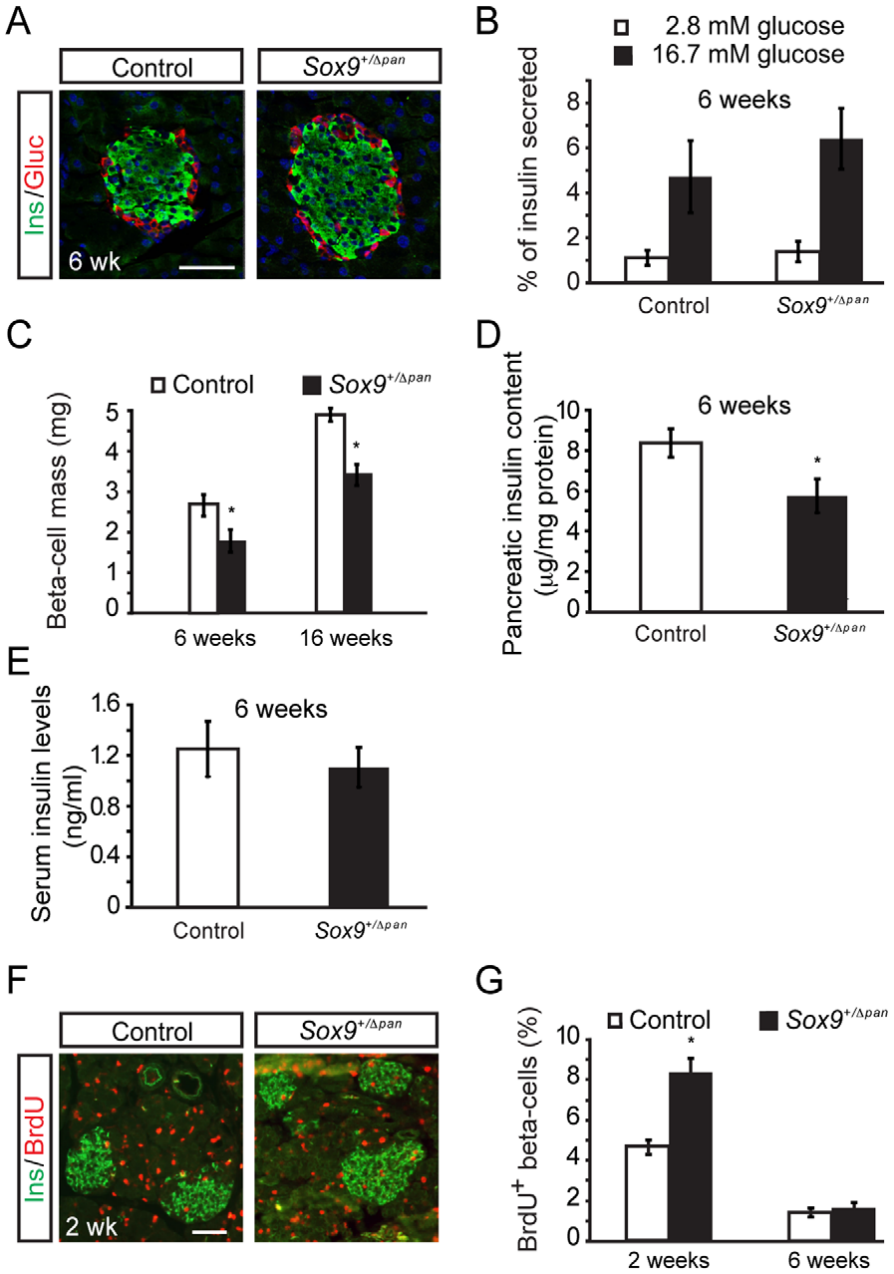
*Sox9^+/Δpan^* mice exhibit compensatory postnatal beta-cell proliferation. (**A**) Immunofluorescence staining of control and *Sox9^+/Δpan^* pancreata for insulin and glucagon reveals normal islet architecture in *Sox9^+/Δpan^* mice. (**B**) Normal glucose stimulated insulin secretion of isolated islets from *Sox9^+/Δpan^* mice (**B**; *n* = 7). (**C**) In *Sox9^+/Δpan^* mice, beta-cell mass is reduced by ∼30% compared to control littermates at 6 and 16 weeks of age (*n* = 3), consistent with pancreatic insulin content in 6-week-old *Sox9^+/Δpan^* mice being 70% that of control siblings (**D**; *n* = 11). (**E**) Serum insulin levels are comparable in *Sox9^+/Δpan^* mice and control littermates (*n* = 11). (**F**, **G**) Beta-cell proliferation, measured by BrdU incorporation into insulin^+^ (Ins^+^) cells, is increased in *Sox9^+/Δpan^* mice at 2 weeks, but not at 6 weeks of age (*n* = 3). Scale bars: 50 µm. Values are shown as mean ± S.E.M.; * = *P*<0.05.

Immunofluorescence analysis revealed that at six weeks of age, while smaller than those of their control siblings, islets of *Sox9^+/Δpan^* mice displayed normal cytoarchitecture, with a central core of insulin^+^ beta-cells surrounded by a mantle composed primarily of glucagon^+^ alpha-cells (**Fig. 7A**). Concordantly, islets isolated from *Sox9*-haploinsufficient mice exhibited normal insulin secretion when stimulated with glucose *in vitro* (**Fig. 7B**). We have previously reported that at e18.5, beta-cell mass in *Sox9^+/Δpan^* mice was 50% reduced compared to control mice [14]. However, by six weeks of age, beta-cell mass in *Sox9* heterozygous mutant mice had risen to 68% that of control littermates, an increase that was maintained at 16 weeks of age (**Fig. 7C**). Consistent with our morphometric data, total pancreatic insulin content in six-week-old *Sox9^+/Δpan^* mice was 70% that in control animals (**Fig. 7D**). Fasting serum insulin levels in *Sox9*-haploinsufficient mice were not significantly different from those of controls (**Fig. 7E**).

The increase in beta-cell mass from 50% to 68% of control mice between e18.5 and six weeks of age suggested that beta-cells of *Sox9^+/Δpan^* mice were capable of adaptive expansion to maintain normoglycemia. This inference was also supported by the fact that these mice did not become overtly diabetic even when metabolically stressed *via* maintenance on a high-fat diet. To determine how and when this increase in beta-cell mass occurred, we measured the proliferation rate of beta-cells in *Sox9^+/Δpan^* mice by assaying for BrdU incorporation at the age of two weeks, which is a dynamic period of islet remodeling and growth in rodents [29], [30]. This analysis revealed that the beta-cell BrdU labeling index in *Sox9^+/Δpan^* mice was twice that in littermate controls, although by six weeks of age, proliferation rates returned to baseline levels (**Fig. 7F,G**). This is consistent with there being no further adaptive increase in beta-cell mass in *Sox9*-haploinsufficient mice beyond six weeks (**Fig. 7C**). Together, our data suggest that while *Sox9*-haploinsufficiency results in a reduced beta-cell complement, adaptive expansion of the beta-cell mass occurs through proliferation to partially restore glucose homeostasis in *Sox9^+/Δpan^* mice.

## Discussion

### Sox9 as a critical regulator of the endocrine differentiation program

As we reported previously [14], *Sox9*-haploinsufficiency leads to a 50% decrease in the numbers of both Ngn3^+^ progenitors and the mature endocrine cells they give rise to. Studies by Lynn *et al.* [15] in the pancreatic ductal mPAC cell line suggested that in addition to *Ngn3*, Sox9 regulates a network of developmental transcription factors upstream of *Ngn3*, including *Hnf6*, *Foxa2*, and *Tcf2*. These findings, reviewed most recently by Pan and Wright [31], while intriguing, demanded further validation in an *in vivo* model. Our finding that neither *Hnf6*, *Foxa2* nor *Tcf2* are dysregulated in the embryonic pancreas following *Sox9* ablation suggests that this same transcriptional network is not conserved in pancreatic progenitor cells *in vivo*.

**Figure 8:**
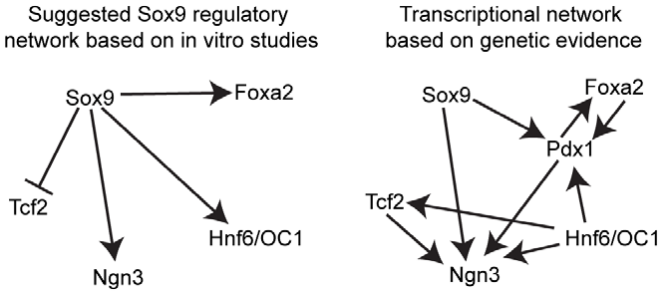
Contrasting the previously suggested Sox9 transcriptional regulatory network during pancreas development to the network based on genetic evidence. (**A**) Proposed Sox9 regulated genes based on *in vitro* evidence [15]. (**B**) Revised model based on this study as well as previous genetic *in vivo* studies [12], [19], [33], [34], [35], [47]. Note that a solid line and arrow does not necessarily indicate direct gene regulation.

Our *in vivo* findings suggest a revised model of the regulatory interactions between key developmental transcription factors in pancreatic progenitor cells (**Fig. 8**). Importantly, our study uncovers a previously unknown role for Sox9 in regulating *Pdx1* expression during the major time window of endocrine cell differentiated in mice. Whether or not Sox9 regulates *Pdx1* expression directly is currently unclear. In the early pancreatic bud, Pdx1 expression is initially maintained upon *Sox9* inactivation *in vivo* [13] and only lost at e12.5, which argues against a role for Sox9 in directly regulating Pdx1 at early pancreatic stages. However, it is possible that Sox9 controls Pdx1 expression at early and later developmental stages by distinct mechanisms and that *Sox9* deletion after e13 has a more immediate effect on Pdx1 expression. *Pdx1* gene transcription is controlled by different enhancers [32] and occupancy of these enhancers by different transcription factors might dynamically change during development. Genome wide DNA occupancy studies for Sox9 in progenitor cells will determine which of the genes that are regulated by Sox9 *in vivo* are direct transcriptional target genes.

Genetic studies have previously shown that similar to Sox9, Hnf6 also functions upstream of Pdx1 [33] and is important for the expression of Ngn3 [34]. Furthermore, Hnf6 has been shown to regulate the expression of Tcf2 (MODY5) [19], a transcription factor that is also required for Ngn3 expression [35]. We show that even though all three factors are co-expressed in the epithelial progenitor cords during the major wave of endocrine cell formation, loss of Sox9 does not affect the expression of Tcf2 or Hnf6. This suggests that Sox9 regulates Pdx1 and Ngn3 expression independent of Tcf2 and Hnf6.

While the requirement for Ngn3 in endocrine development is well established, the network of transcription factors required for its activation has not been comprehensively identified, nor their interactions clearly defined. Here, we reveal that Sox9 is required for proper *Ngn3* induction through several possible mechanisms. Previous observations led us to suggest a mechanism through which Sox9 induces *Ngn3* expression. Wang *et al.* have recently shown that Ngn3 expression in pancreatic progenitors must attain a critical high “Ngn3^Hi^” threshold level to initiate an endocrine developmental program in those cells [36]. It is conceivable that Sox9 activates *Ngn3* in a dosage-sensitive manner such that a Sox9^Hi^ expression level is crucial for activation of a Ngn3^Hi^ expression level sufficient to induce endocrine differentiation. In support of this hypothesis, we observed a significant reduction in the number of Ngn3^Hi^ cells in Sox9-haploinsufficient mice [14]. Additionally, Pdx1, which we show is expressed in a Sox9-dependent manner, has also been reported to activate *Ngn3* in coordination with Hnf6 [12]. Thus, Sox9 contributes to acquisition of the Ngn3^Hi^ state by two mechanisms: (1) by directly activating *Ngn3* and (2) by reinforcing *Ngn3* expression through positive regulation of *Pdx1*. Since no functional role has thus far been demonstrated for Sox9 in endocrine cell maturation [14], those progenitors that attain sufficiently high *Ngn3* expression to initiate an endocrine program should differentiate normally, so that, albeit reduced in numbers, the adult beta-cells of *Sox9*-haploinsufficient pancreata are mature and fully functional. Consistent with this notion, we found that islets from *Sox9*-haploinsufficient mice exhibit normal GSIS *in vitro*.

### The role of *Sox9* in maintaining adult glucose homeostasis

In humans, *Sox9*-haploinsufficiency is associated with the syndrome campomelic dysplasia (CD). Although pancreatic islet abnormalities have been reported in neonatal CD cases [37], the early lethality of the condition precludes the manifestation of metabolic defects. However, as pancreas-specific expression of *Sox9* is governed by enhancer region E1, located approximately 28 kb 5′ of the transcription initiation site in humans [38], it is conceivable that enhancer-specific *Sox9* mutations could cause a diabetic phenotype in humans. The viability of mice displaying pancreas-specific heterozygous loss of *Sox9* afforded us the unique opportunity of studying the effects of reduced *Sox9* dosage on pancreatic endocrine function in adult mice, to test whether *Sox9* is a potential MODY or diabetes susceptibility gene.

While the majority of MODY genes play important functional roles in adult beta-cells [16], [17], [27], [39], [40], *Tcf2*/MODY5 is the exception. Like Sox9, Tcf2 is excluded from beta-cells and instead, is expressed in the pancreatic progenitor epithelium during embryonic development, becoming restricted to duct and centroacinar cells in adulthood [3], [4]. Pancreatic hypoplasia, pancreatic atrophy, defective expression of Glut2, and impaired insulin secretion have been reported in human cases of *Tcf2* mutation [35], [41]. This suggests that dysregulation of factors not expressed in beta-cells but expressed in their precursors, can cause diabetes independently of beta-cell dysfunction, due presumably to pancreatic endocrine dysgenesis. While we found no evidence for pancreatic Tcf2 expression being Sox9-dependent, our data suggest that *Sox9*-haploinsufficiency might itself manifest in a MODY phenotype. Furthermore, the fact that Sox9 regulates expression of the MODY4 gene *Pdx1* during development raises the possibility that the diabetic phenotype caused by heterozygous *Pdx1* mutations [7] could in part be attributable to impaired beta-cell development.

While we found a 50% reduction in *Sox9* gene dosage to cause glucose intolerance in mice, *Sox9^+/Δpan^* mice did not develop overt diabetes. This raises the question of how severely reduced pancreatic *Sox9* levels might affect glucose homeostasis in humans. Interestingly, attempts to generate mouse models for human MODY have revealed that mutations in mouse homologues of human MODY genes do not always phenocopy the human condition. While *Tcf2*-haploinsufficient humans display diabetes and severe pancreatic endocrine defects [42], [43], partially mirroring our findings in *Sox9^+/Δpan^* mice, *Tcf2^+/−^* mice do not display diabetic symptoms [44]. Additionally, the MODY3 phenotype is not recapitulated in *Hnf1a^+/−^* mice, although *Hnf1a^−/−^* mice do become diabetic [17]. One possible explanation for the discrepancy between the metabolic effects of MODY gene mutations in mouse and humans is that the beta-cells of mice are able to better compensate for the reduction in gene dosage than those of humans. Consistent with this idea, we observed compensatory beta-cell proliferation in *Sox9^+/Δpan^* mice, which led to a significant, albeit not complete, recovery of beta-cell mass during the early postnatal period. Because human beta-cells have a much lower proliferative capacity than murine beta-cells [45], it is predicted that reduced Sox9 levels in humans will have more profound effects on glucose homeostasis than observed in *Sox9*-haploinsufficient mice. Although to this point *Sox9* has not emerged as a type 2 diabetes susceptibility gene from genome-wide association studies (GWAS) [46], other genes with known roles in human diabetes, such as *Pdx1*, also failed to show an association with type 2 diabetes in GWAS. Additional studies are therefore required to determine whether mutations in *Sox9* are associated with diabetes in humans.
